# 

**Published:** 1885-11

**Authors:** 


### CONTENTS:

Art. I—The Georgia State Dental Society ........................................289
Art. II—The Nerve Centers and the Teeth, By J. Smith Dodge, Jr., M. D., D. D. S., 299
Art. III—A Factor in Tooth-Preservation, By C. N. Pierce, D. D. S. ..............305
Art. IV—Etiology of Dental Caries, By Richard White, L. D. S ..................318
Art. V—Food of Infants, By Fred. Hooper Hayes, D. D. S.........................323
Art. VI—The New Georgia Dental Law .........................................326
Art. VII—Virginia State Dental Association.......................................328
Art. VIII—American Academy of Dental Science...................................329

### EDITORIAL, ETC.

Ninth International Medical Congress.............................................330

### BIBLIOGRAPHICAL.

A Series of Questions Pertaining to the Curriculum of the Dental Student, By Ferdinand J. S. Gorgas, A. M., M. D., D. D. S., of the University of Maryland.............................................333
Quiz Questions, By Prof. J. F. Flagg, D. D. S.....................................333
Dental Bibliography, By C. G. Crowley...........................................334
The Physicians Visiting List (Lindsay & Blakiston)...............................334

### MONTHLY SUMMARY.

A Peculiar Case.................................................................335

Oxy-Phosphate...................................................................336

